# Synergistic anti-*Campylobacter jejuni* activity of fluoroquinolone and macrolide antibiotics with phenolic compounds

**DOI:** 10.3389/fmicb.2015.01129

**Published:** 2015-10-13

**Authors:** Euna Oh, Byeonghwa Jeon

**Affiliations:** School of Public Health, University of Alberta, EdmontonAB, Canada

**Keywords:** *Campylobacter jejuni*, fluoroquinolones, macrolides, phenolic compound, synergism

## Abstract

The increasing resistance of *Campylobacter* to clinically important antibiotics, such as fluoroquinolones and macrolides, is a serious public health problem. The objective of this study is to investigate synergistic anti-*Campylobacter jejuni* activity of fluoroquinolones and macrolides in combination with phenolic compounds. Synergistic antimicrobial activity was measured by performing a checkerboard assay with ciprofloxacin and erythromycin in the presence of 21 phenolic compounds. Membrane permeability changes in *C. jejuni* by phenolic compounds were determined by measuring the level of intracellular uptake of 1-*N*-phenylnaphthylamine (NPN). Antibiotic accumulation assays were performed to evaluate the level of ciprofloxacin accumulation in *C. jejuni*. Six phenolic compounds, including *p*-coumaric acid, sinapic acid, caffeic acid, vanillic acid, gallic acid, and taxifolin, significantly increased the susceptibility to ciprofloxacin and erythromycin in several human and poultry isolates. The synergistic antimicrobial effect was also observed in ciprofloxacin- and erythromycin-resistant *C. jejuni* strains. The phenolic compounds also substantially increased membrane permeability and antibiotic accumulation in *C. jejuni*. Interestingly, some phenolic compounds, such as gallic acid and taxifolin, significantly reduced the expression of the CmeABC multidrug eﬄux pump. Phenolic compounds increased the NPN accumulation in the *cmeB* mutant, indicating phenolic compounds may affect the membrane permeability. In this study, we successfully demonstrated that combinational treatment of *C. jejuni* with antibiotics and phenolic compounds synergistically inhibits *C. jejuni* by impacting both antimicrobial influx and eﬄux.

## Introduction

*Campylobacter jejuni* is one of the leading bacterial causes of human gastroenteritis worldwide (Butzler, 2004). It is estimated that *Campylobacter* accounts for approximately 400–500 million infection cases worldwide per year (Ruiz-Palacios, 2007). *C. jejuni* inhabits the gastrointestinal tracts of poultry as a commensal microorganism; thus, the consumption of undercooked poultry is the most frequent cause of human infections with *C. jejuni* (Ruiz-Palacios, 2007). *C. jejuni* can also spread by cross-contamination and during inadequate storage (Cogan et al., 1999; Luber et al., 2006). Particularly, the dissemination of foodborne pathogens via hands and food-contact surfaces of food processing equipment has been well documented by a number of researchers (Kusumaningrum et al., 2003; Van Asselt et al., 2008).

For the clinical treatment of serious campylobacteriosis, fluoroquinolones and macrolides are drugs of choice (Luangtongkum et al., 2009). However, the increasing resistance to the clinically important antibiotics in *C. jejuni* is widespread worldwide and significantly compromised the effectiveness of current antibiotic chemotherapy, frequently leading to severe patient outcomes, such as prolonged hospitalization, high mortality, and treatment failure (Helms et al., 2005). For example, ciprofloxacin resistance is approximately 92% in *C. jejuni* isolates from raw chicken in South Korea (Han et al., 2007) and even 100% in clinical isolates from children in Thailand (Serichantalergs et al., 2007). Among the antibiotic resistance determinants in *C. jejuni*, the multidrug eﬄux pump CmeABC is most well-characterized (Lin et al., 2002; Martinez and Lin, 2006). CmeABC is a resistance-nodulation-cell division (RND) type of multidrug eﬄux pump and consists of three protein components, including periplasmic fusion protein (i.e., CmeA), an inner membrane drug transporter (i.e., CmeB), and outer membrane protein (i.e., CmeC) (Lin et al., 2002).

Phenolic compounds are a group of secondary metabolites present in a wide range of plants. The beneficial health effects of dietary supplementation of phenolic compounds have been reported in a significant number of studies, regarding their anti-oxidation, anti-cancer, anti-diabetic, and anti-aging activities (Pandey and Rizvi, 2009). Additionally, some phenolic compounds possess antimicrobial activities against various pathogenic bacteria (Daglia, 2012), including *C. jejuni* (Klancnik et al., 2012). The CmeABC eﬄux pump plays an important role in *C. jejuni* resistance to phenolic compounds (Klancnik et al., 2012). Recently, we also demonstrated that some phenolic compounds exhibit anti-*C. jejuni* activity (Oh and Jeon, 2015). In this study, we investigated the anti-*Campylobacter* activity of combinational treatment of phenolic compounds with antibiotics of clinical importance for the treatment of human campylobacteriosis.

## Materials and Methods

### Bacterial Strains and Culture Conditions

*Campylobacter jejuni* NCTC 11168 is the wild-type strain (Parkhill et al., 2000), and *C. jejuni* CR64 and ER641 are NCTC 11168 derivatives resistant to ciprofloxacin and erythromycin, respectively. Briefly, *C. jejuni* CR64 and ER641 were generated by increasing the antibiotic concentrations in culture media from 0.1 μg ml^-1^ to 64 μg ml^-1^. We chose resistant *C. jejuni* strains by growing on MH agar plates supplemented with 64 μg ml^-1^ of ciprofloxacin and erythromycin, and mutations in *gyrA* and 23S rRNA, respectively, were observed by sequencing (data not shown). *C. jejuni* P1 and P2 were isolated from retail poultry meats. *C. jejuni* HCJ4132 and HCJ2316 are human isolates, a kind gift from Dr. Monika Keelan (University of Alberta). A *cmeB* mutant of *C. jejuni* NCTC 11168 was reported previously (Akiba et al., 2006). *C. jejuni* strains were routinely grown on Mueller–Hinton (MH) medium at 42°C under microaerobic conditions (5% O_2_, 10% CO_2_, and 85% N_2_).

### Checkerboard Titration Assay

The MICs of ciprofloxacin and erythromycin were measured in the presence of phenolic compounds, including 13 phenolic acids (*p*-coumaric acid, caffeic acid, sinapic acid, ferulic acid, cinnamic acid, vanillic acid, salicylic acid, gallic acid, benzoic acid, *p*-hydroxybenzoic acid, tannic acid, protocatechuic acid, and syringic acid) and eight flavonoids [epigallocatechin gallate, (-)-epicatechin, morin, quercetin, chrysin, naringenin, hesperidin, and taxifolin] with *C. jejuni* NCTC 11168. For *C. jejuni* P1, P2, HCJ4132, HCJ2316, CR64, and ER641 strains, the MICs of ciprofloxacin and erythromycin were measured in combination with *p*-coumaric acid, sinapic acid, caffeic acid, vanillic acid, gallic acid, and taxifolin. All these phenolic compounds were purchased from Sigma–Aldrich (St. Louis, MO, USA). The checkerboard titration assay was performed as described previously (Hsieh et al., 1993). Briefly, antibiotics were twofold serially diluted on each column, and phenolic compounds were twofold diluted on each row. The concentrations of phenolic compounds and antibiotics started from their MICs. *C. jejuni* suspension (ca., 10^5^ CFU per well) was added, and the plate was incubated at 42°C for 18 h under microaerobic conditions.

### Membrane Permeability Test

Membrane permeability assay was performed as described elsewhere (Helander and Mattila-Sandholm, 2000). Briefly, overnight cultures of *C. jejuni* strains were diluted in MH broth to an OD_600_ of 0.07. The *C. jejuni* suspensions in MH broth were grown at various concentrations of phenolic compounds, including 1–128 μg ml^-1^ of *p*-coumaric acid, 0.5–64 μg ml^-1^ of gallic acid, or 0.25–32 μg ml^-1^ of taxifolin, at 42°C for 18 h under microaerobic conditions. The bacterial cells were harvested by centrifugation and washed twice with PBS (pH 7.4) and resuspended with 100 μl PBS containing 10 μM 1-*N*-phenylnaphthylamine (NPN) for 5 min. EDTA was used as a positive control for permeability (Hancock and Wong, 1984). Fluorescence was measured at 335/405 nm (excitation/emission) with FLUOstar Omega (BMG Labtech, Germany).

### Ciprofloxacin Accumulation Assay

A ciprofloxacin accumulation assay was performed according to a method described previously (Jeon et al., 2011). Briefly, *C. jejuni* NCTC 11168 was grown overnight to around the late log phase in MH broth with 1–128 μg ml^-1^ of *p*-coumaric acid, gallic acid, and taxifolin. The bacterial cells were harvested and washed once with PBS (pH 7.4), and then resuspended in PBS. The phenolic compounds did not affect the growth of *C. jejuni* since the MICs of *p*-coumaric acid, gallic acid, and taxifolin are 1024 μg ml^-1^, 512 μg ml^-1^, and 256 μg ml^-1^, respectively (Oh and Jeon, 2015). These samples were incubated at 37°C for 10 min, and ciprofloxacin was added to a final concentration of 10 μg ml^-1^. After incubation at room temperature for 20 min, the bacterial suspension (0.5 ml) was diluted with 2.5 ml of ice-cold PBS and centrifuged at 6000 ×*g*, 4°C for 10 min. After washing twice with ice-cold PBS, the harvested cells were resuspended in 0.2 ml of 0.1 M glycine hydrochloride (pH 3.0) and incubated at room temperature with shaking for 16 h. The supernatant was obtained by centrifugation at 15000 ×*g* for 10 min, and fluorescence was measured at 279/447 nm (excitation/emission) with FLUOstar Omega (BMG Labtech).

### *P_cmeABC_*::*lacZ* Promoter Fusion Assay

*C. jejuni* NCTC 11168 including *P_cmeABC_*::*lacZ* was constructed previously (Hwang et al., 2012). *C. jejuni* was grown overnight on MH agar including kanamycin (50 μg ml^-1^) at 42°C under microaerobic conditions. *C. jejuni* was harvested and diluted in MH broth to an OD_600_ of 0.07. *C. jejuni* was grown at 42°C for 5 h under microaerobic conditions and then was exposed to 1 μg ml^-1^of each phenolic compound for 2 h. β-galactosidase assays were carried out as described in a previous study (Kim et al., 2015).

### Western Blot Analysis

*Campylobacter jejuni* NCTC 11168 was grown on MH agar plates and harvested in fresh MH broth as described above. Broth culture of *C. jejuni* were grown at 42°C for 7 h under microaerobic conditions with shaking (200 rpm) in present of 1 μg ml^-1^ of phenolic compounds, including *p*-coumaric acid, sinapic acid, caffeic acid, vanillic acid, gallic acid, and taxifolin. Western blot analysis was performed as described previously (Lin et al., 2002) with a 10% polyacrylamide gel in Tris-Tricine buffer. The polyclonal antibody against CmeA is a kind gift from Dr. Qijing Zhang (Iowa State University, USA).

## Results

### Synergistic Antimicrobial Effect of Phenolics with Antibiotics

Twenty one phenolic compounds, including 13 phenolic acids and eight flavonoids, were screened to examine synergistic antimicrobial activity with ciprofloxacin and erythromycin. The MICs of the phenolic compounds were already determined in our previous study and are mostly greater than 256 μg ml^-1^ (Oh and Jeon, 2015). Synergistic antimicrobial activity was observed in six phenolic compounds, including five phenolic acids (*p*-coumaric acid, sinapic acid, caffeic acid, vanillic acid, gallic acid) and one flavonoid (taxifolin; **Table 1** and Supplementary Tables S1–S7). For example, 8 μg ml^-1^ of the six phenolic compounds resulted in approximately 4–32-fold reduction in the MICs of ciprofloxacin and erythromycin in five different *C. jejuni* strains, including three human isolates (NCTC 11168, HCJ 4132, and HCJ 2316) and two poultry isolates (P1 and P2; **Table 1** and Supplementary Tables S3–S7). A further MIC reduction was observed at increased concentrations of phenolic compounds; the MIC of ciprofloxacin was reduced by 64-fold in combination with 128 μg ml^-1^ *p*-coumaric acid, vanillic acid, taxifolin, and 256 μg ml^-1^ sinapic acid, vanillic acid, and gallic acid in *C. jejuni* NCTC11168 (Supplementary Table S3). The fractional inhibitory concentration (FIC) index is frequently used to evaluate if the reaction is synergistic or not; the FIC values of 0.5 or less indicate synergy (Rand et al., 1993). The FIC values also confirmed that the increased antimicrobial activity is synergistic in *C. jejuni* NCTC 11168 (Supplementary Table S9). These results clearly show that some phenolic compounds synergistically enhance the antimicrobial activity of ciprofloxacin and erythromycin against *C. jejuni.*

**Table 1:** Synergistic antimicrobial activity of phenolic compounds with ciprofloxacin and erythromycin in various *Campylobacter jejuni* strains.

Ciprofloxacin MIC (μg ml^-1^) in the presence of phenolic compounds						
	*C. jejuni* 11168	*C. jejuni* CR64	*C. jejuni* P1	*C. jejuni* P2	*C. jejuni* HCJ4132	*C. jejuni* HCJ2316
*p*-Coumaric acid	0.063 (8)	8 (8)	0.004 (32)	0.125 (8)	0.016 (16)	0.016 (16)
Sinapic acid	0.063 (8)	8 (8)	0.016 (8)	0.063 (16)	0.031 (8)	0.031 (8)
Caffeic acid	0.125 (4)	16 (4)	0.031 (4)	0.125 (8)	0.031 (8)	0.031 (8)
Vanillic acid	0.063 (8)	8 (8)	0.008 (16)	0.25 (4)	0.031 (8)	0.031 (8)
Gallic acid	0.063 (8)	8 (8)	0.008 (16)	0.125 (8)	0.016 (16)	0.031 (8)
Taxifolin	0.063 (8)	2 (32)	0.008 (16)	0.063 (16)	0.031 (8)	0.031 (8)
Ciprofloxacin without phenolic compounds	0.5 (1)	64 (1)	0.125 (1)	1 (1)	0.25 (1)	0.25 (1)

**Erythromycin MIC (μg ml^-1^) in the presence of phenolic compounds**						
	***C. jejuni* 11168**	***C. jejuni* ER641**	***C. jejuni* P1**	***C. jejuni* P2**	***C. jejuni* HCJ4132**	***C. jejuni* HCJ2316**

*p*-Coumaric acid	0.125 (4)	16 (4)	0.063 (8)	0.125 (8)	0.25 (1)	0.063 (4)
Sinapic acid	0.063 (8)	16 (4)	0.063 (8)	0.125 (8)	0.125 (2)	0.063 (4)
Caffeic acid	0.125 (4)	16 (4)	0.063 (8)	0.25 (4)	0.125 (2)	0.125 (2)
Vanillic acid	0.125 (4)	16 (4)	0.125 (4)	0.25 (4)	0.125 (2)	0.125 (2)
Gallic acid	0.063 (8)	16 (4)	0.125 (4)	0.125 (8)	0.063 (4)	0.063 (4)
Taxifolin	0.031 (16)	4 (16)	0.031 (16)	0.063 (16)	0.063 (4)	0.031 (8)
Erythromycin without phenolic compounds	0.5 (1)	64 (1)	0.5 (1)	1 (1)	0.25 (1)	0.25 (1)

*The results are representative of three independent experiments. The table indicates the levels of MIC and fold changes in parenthesis. The concentration of phenolic compounds is 8 μg ml^-*1*^. Synergistic effects in a wide range of phenolic concentrations are listed in Supplementary Tables S3–S8.*

### Synergistic Antimicrobial Activity of Phenolic Compounds against Antibiotic-Resistant *C. jejuni*

To further investigate the synergistic antimicrobial activity of phenolic compounds against antibiotic-resistant *C. jejuni*, we performed a checkerboard assay with *C. jejuni* CR64 and ER641 strains; *C. jejuni* CR64 is fluoroquinolone-resistant due to a C257T mutation in *gyrA*, and *C. jejuni* ER641 is resistant to erythromycin because of an A2074C mutation in 23S rRNA. Interestingly, a substantial synergistic antimicrobial effect was also observed in the resistant strains (Supplementary Table S8). Particularly, 8 μg ml^-1^ taxifolin reduced the MICs of ciprofloxacin and erythromycin by 32- and 16-fold, respectively (**Table 1**). The synergistic antimicrobial activity was dependent on the concentration of phenolic compounds (Supplementary Table S8). These findings exhibit that phenolic compounds sensitizes resistant *C. jejuni* strains to antibiotics.

### Increased Membrane Permeability by Phenolic Compounds

**FIGURE 1:**
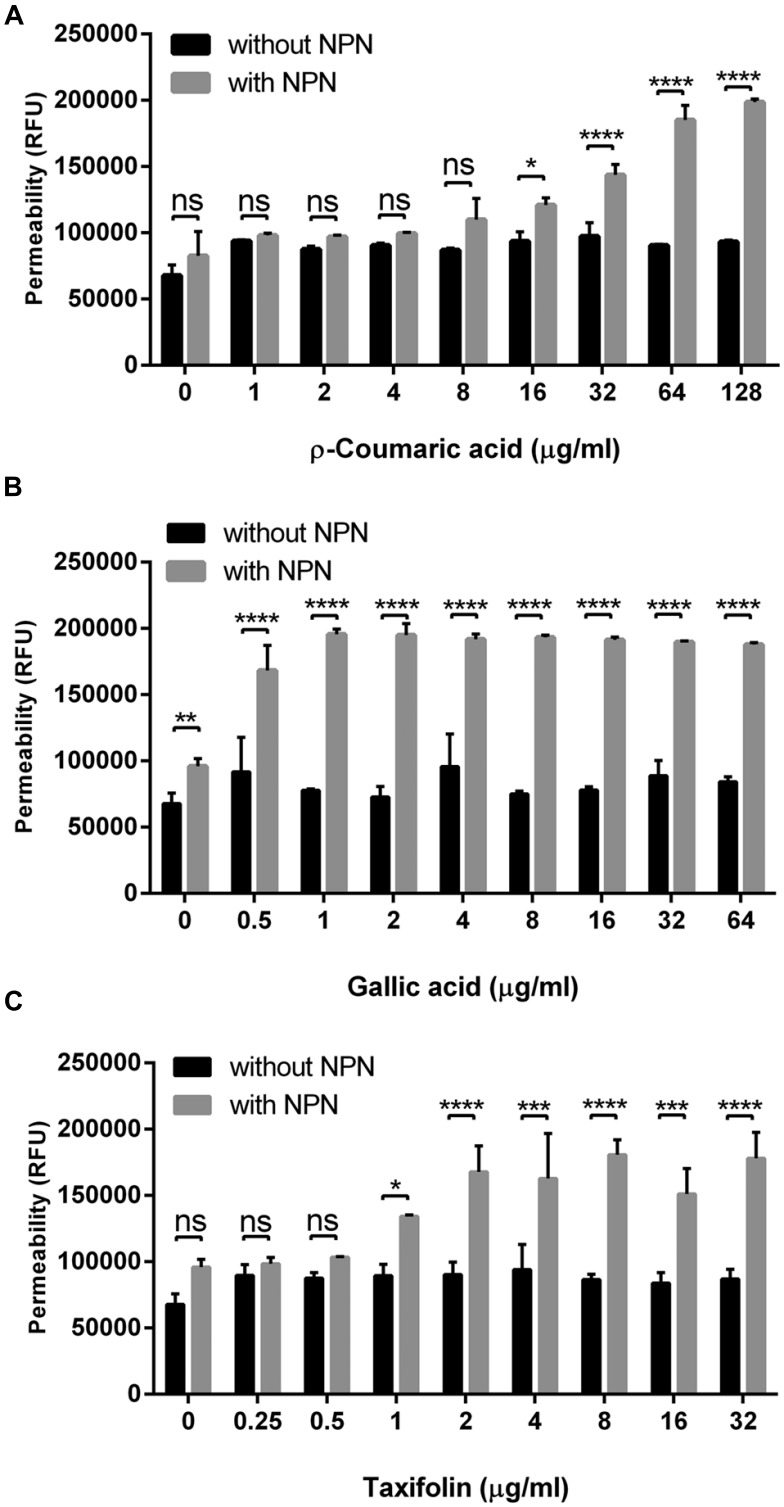
**Changes in the membrane permeability of *Campylobacter jejuni* by *p*-coumaric acid **(A)**, gallic acid **(B)**, and taxifolin **(C)**.** The assay was carried out with 10 μM 1-*N*-phenylnaphthylamine (NPN). Fluorescence from samples with NPN treatment reflects permeability increase, and RFU from samples without NPN shows the background fluorescence level. The results show the means and standard deviations of triplicate samples in a single experiment. The experiment was repeated three times, and all the experiments produced similar results. Statistical significance was determined with two-way ANOVA using GraphPad Prism 6 (GraphPad Software Inc., USA). ns: *P* > 0.5, ^∗^*P* < 0.05, ^∗∗^*P* < 0.01, ^∗∗∗^*P* < 0.001, ^∗∗∗∗^*P* < 0.0001.

Since some phenolic compounds have been reported to affect membrane permeability in other Gram-negative bacteria (Nohynek et al., 2006; Borges et al., 2013), we investigated if phenolic compounds would change membrane permeability in *C. jejuni* NCTC 11168. Two phenolic acids (i.e., *p*-coumaric acid and gallic acid) and one flavonoid (i.e., taxifolin), which consistently exhibited synergistic anti-*Campylobacter* activity with ciprofloxacin and erythromycin (**Table 1** and Supplementary Table S3), were chosen for the permeability assay with NPN. The NPN uptake rates were increased in a manner dependent on the concentration of phenolic compounds (**Figure 1**). Membrane permeability was significantly increased at concentrations as low as 0.5 μg ml^-1^ gallic acid, 1 μg ml^-1^ taxifolin and 16 μg ml^-1^ *p*-coumaric acid (**Figure 1**). A few other phenolic compounds, such as caffeic acid, vanillic acid, and sinapic acid, also increased the membrane permeability in *C. jejuni*. However, other phenolic compounds did not show changes in membrane permeability in *C. jejuni* (Supplementary Figure S1). EDTA, a permeability enhancement agent, was used as a positive control; a high concentration (0.5 mM) of EDTA increased membrane permeability. The six phenolic compounds (i.e., *p*-coumaric acid, gallic acid, taxifolin, caffeic acid, vanillic acid, and sinapic acid) enhanced the membrane permeability at an EDTA concentration (0.02 mM) that did not affect membrane permeability (Supplementary Figure S2). This confirmed the effect of phenolic compounds on the alteration of membrane permeability. These findings indicated that some phenolic compounds may significantly affect membrane permeability in *C. jejuni*.

### Increased Antibiotic Accumulation by Phenolic Compounds

**FIGURE 2:**
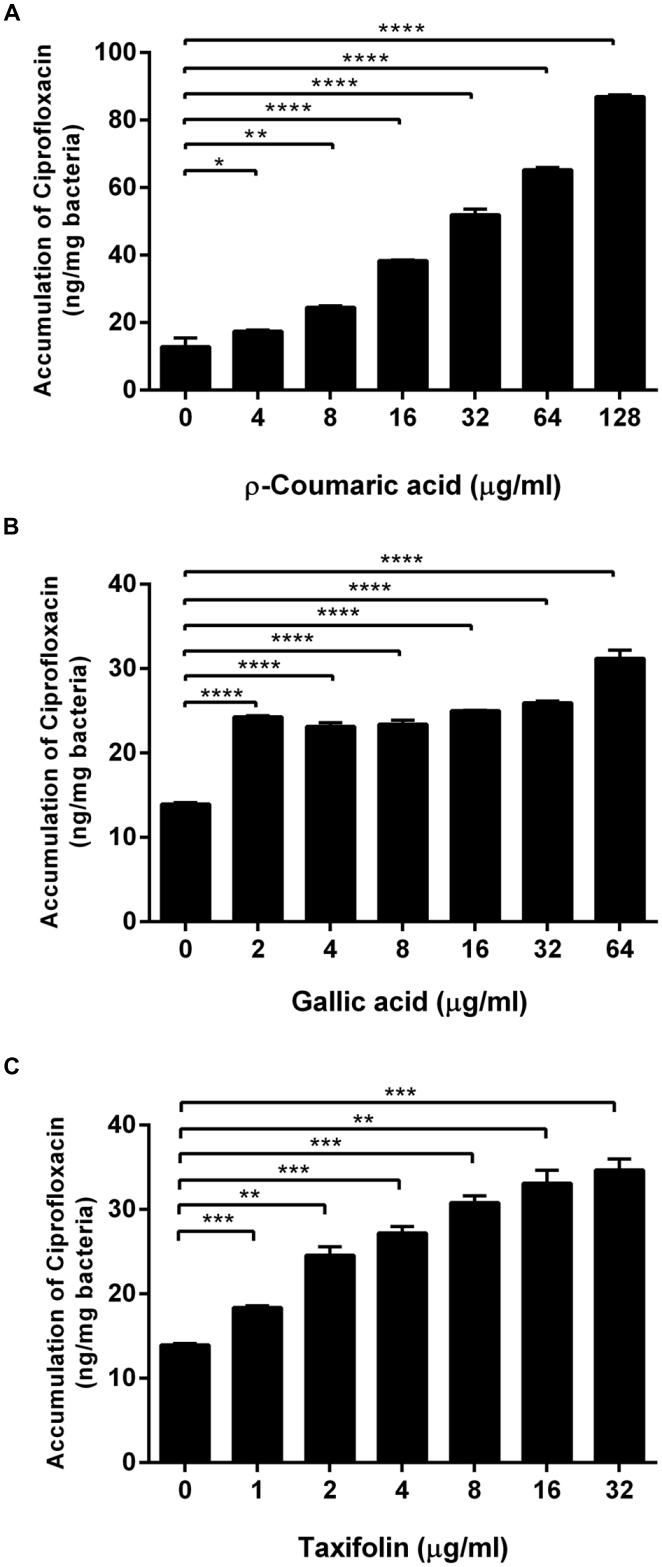
**Increased accumulation of ciprofloxacin in *C. jejuni* by *p*-coumaric acid **(A)**, gallic acid **(B)**, and taxifolin **(C)**.** The results show the means and standard deviations of triplicate samples in a single experiment. Similar results were obtained in three independent experiments. Statistical analysis was carried out with the Student’s *t*-test using GraphPad Prism 6 (GraphPad Software Inc.). ^∗^*P* < 0.05, ^∗∗^*P* < 0.01, ^∗∗∗^*P* < 0.001, ^∗∗∗∗^*P* < 0.0001.

Since we observed that phenolic compounds increased membrane permeability in *C. jejuni* NCTC 11168 (**Figure 1**), we determined if phenolic compounds may also increase antibiotic accumulation in *C. jejuni*. An antibiotic accumulation assay was performed with ciprofloxacin in the presence of *p*-coumaric acid, gallic acid, and taxifolin. Interestingly, ciprofloxacin accumulation in *C. jejuni* was substantially enhanced by the phenolic compounds. For example, 8 μg ml^-1^ *p*-coumaric acid, and 2 μg ml^-1^ gallic acid and taxifolin increased the level of ciprofloxacin accumulation approximately by twofold, compared to a non-treated control (**Figure 2**). The results show that phenolic compound treatment enhances antibiotic accumulation in *C. jejuni*.

### Changes in CmeABC Expression by Phenolic Compounds

**FIGURE 3:**
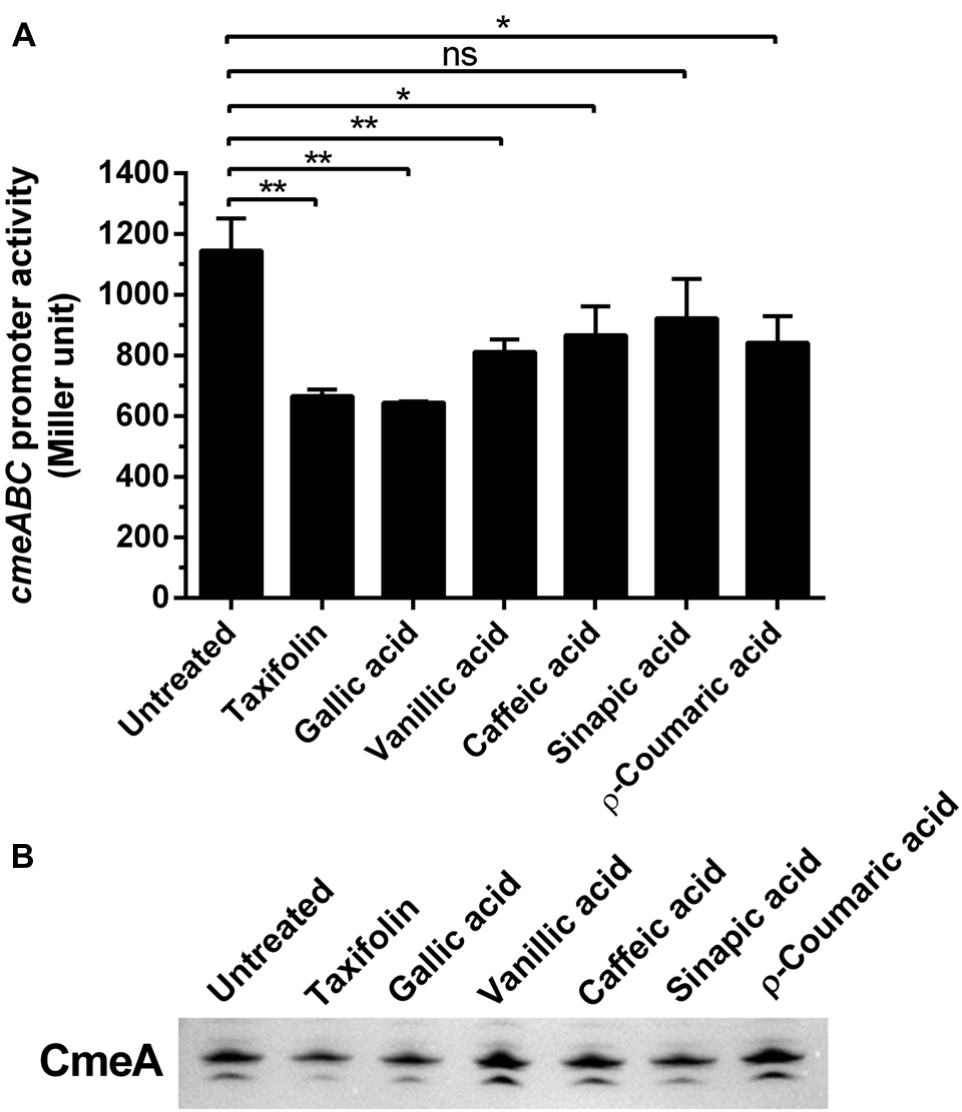
**Changes in the expression levels of CmeABC by phenolic compounds. (A)** Transcriptional levels of *cmeABC* determined by a *P_cmeABC_*::*lacZ* promoter fusion system with 1 μg ml^-1^ phenolic compounds. The results exhibit the means and standard deviations of triplicate samples in a single experiment. The experiment was repeated three times. Statistical analysis was performed with Student’s *t*-test using GraphPad Prims 6 (GraphPad Software Inc.). ns: *P* > 0.5, ^∗^*P* < 0.05, ^∗∗^*P* < 0.01. **(B)** The level of CmeA protein was demonstrated by western blotting with phenolic compounds. The CmeABC eﬄux pump was differentially expressed by phenolic compounds. Phenolic compounds (1 μg ml^-1^) were treated on each sample.

Since CmeABC is a key resistance determinant in *C. jejuni*, we examined if the phenolic compounds would affect the expression of CmeABC in *C. jejuni*. First, we determined the level of *cmeABC* transcription in the presence of phenolic compounds by using a *P_cmeABC_*::*lacZ* fusion. Interestingly, the phenolic compounds overall reduced *cmeABC* transcription. Particularly, gallic acid and taxifolin resulted in most significant reduction in the level of *cmeABC* transcription (**Figure 3A**). Western blotting results also showed that phenolic compounds, especially gallic acid and taxifolin, decreased the translational levels of CmeA (**Figure 3B**). As to the other phenolic compounds, the translational levels were not consistent with the transcriptional levels. For example, vanillic acid, caffeic acid, and *p*-coumaric reduced the level of *cmeABC* transcription; however, the reduction was not observed at the translational level in our repeated experiments. These results suggest some phenolic compounds, such as gallic acid and taxifolin, affect CmeABC expression.

### Combinational Effects of Phenolic Compounds on CmeABC and Membrane Permeability

**FIGURE 4:**
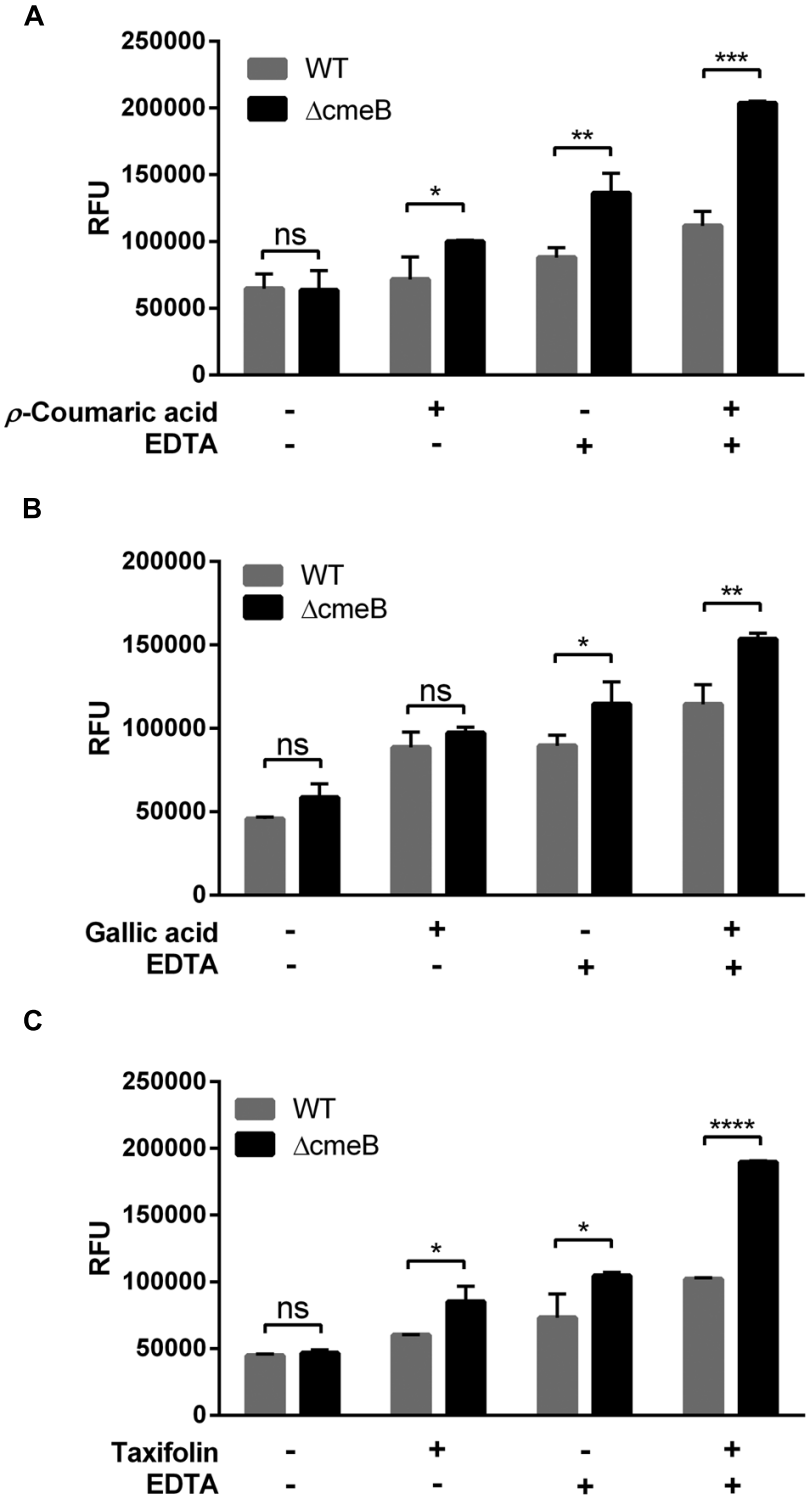
**Membrane permeability changes in *C. jejuni* NCTC 11168 (WT) and its isogenic *cmeB* mutant by *p*-coumaric acid **(A)**, gallic acid **(B)**, and taxifolin **(C)**.** The NPN permeabilization assay was performed with 10 μM NPN. In the assay, 1 μg ml^-1^ phenolic compounds and 0.05 mM EDTA were used. Similar results were produced in three independent experiments. Statistical analysis was carried out with Student’s *t*-test using GraphPad Prism 6 (GraphPad Software INC.). ns: *P* > 0.5, ^∗^*P* < 0.05, ^∗∗^*P* < 0.01, ^∗∗∗^*P* < 0.001, ^∗∗∗∗^*P* < 0.0001.

Some phenolic compounds significantly increased the membrane permeability (**Figure 1**) and reduced CmeABC expression (**Figure 3**). However, it was not clear how phenolic compounds affect membrane permeability interactively via CmeABC and the membrane integrity in *C. jejuni*. To elucidate this, we performed a membrane permeability assay with a *cmeB* mutant in the presence of phenolic compounds and/or EDTA. Taxifolin and *p*-coumaric acid made the *cmeB* mutant accumulate more NPN than WT (i.e., *C. jejuni* NCTC 11168; **Figure 4**). The NPN accumulation was increased in the *cmeB* mutant by EDTA and further by combination of EDTA with phenolic compounds (**Figure 4**). Although phenolic compounds differentially affected the NPN accumulation between WT and the *cmeB* mutant, phenolic compounds commonly increased the NPN accumulation in both WT and the *cmeB* mutant. These results suggest that the increased NPN accumulation by phenolic compounds would be associated with both the CmeABC drug eﬄux pump and the alteration in membrane permeability.

## Discussion

By screening 21 phenolic compounds, in this study, we identified six phenolic compounds that substantially increased the antimicrobial activity of ciprofloxacin and erythromycin against several *C. jejuni* strains from humans and poultry (**Table 1** and Supplementary Tables S1–S7), including strains with antibiotic resistance (**Table 1** and Supplementary Table S8). Although the level of MIC reduction slightly varied depending on the strain, the phenolic compounds significantly sensitized *C. jejuni* to antibiotics. The synergism has been reported in some other bacteria. Green tea extracts containing polyphenols significantly decreased the MIC of oxacillin in *Staphylococcus aureus* (Cho et al., 2008). Gallic acid increases the antimicrobial efficacy of amikacin, norfloxacin, gentamicin, and sulfamethoxazole in *Escherichia coli* (Neyestani et al., 2007). The synergistic antimicrobial activity of phenolics has been demonstrated even in an animal model. Treatment of chronic bacterial prostatitis with ciprofloxacin in combination with catechin, a flavonoid, significantly decreases the growth of *E. coli* even in animal experiments using rats (Lee et al., 2005). To the best of our knowledge, this is the first report about synergistic antimicrobial activity between antibiotics and phenolic compounds in *C. jejuni*.

The synergistic antimicrobial activity of phenolic compounds is associated with the alteration in membrane permeability and antibiotic accumulation in *C. jejuni* (**Figures 1** and **2**). Borges et al. (2013) reported that gallic acid relatively at high concentrations (i.e., >100 μg ml^-1^) increases membrane permeability in Gram-negatives and -positives, such as *E. coli, Pseudomonas aeruginosa*, and *Listeria monocytogenes*. Compared to these pathogens, however, gallic acid effectively reduced permeability in *C. jejuni* at significantly low concentrations. For example, 2 μg ml^-1^ gallic acid resulted in twofold increases in permeability (**Figure 1B**) and antibiotic accumulation (**Figure 2B**), and an eightfold reduction in the MIC of ciprofloxacin (**Table 1**), suggesting that *C. jejuni* is more likely to be permeabilized by phenolics than these pathogenic bacteria. Although its molecular mechanism remains unknown, presumably, it could be because *C. jejuni* possesses lipooligosaccharide (LOS), not lipopolysaccharide (LPS). Surface polysaccharides, particularly LPS, constitute a critical permeability barrier to the entry of hydrophobic compounds into the cell (Vaara, 1992); in our previous study, we also demonstrated LOS is a permeability barrier to phenolic compounds in *C. jejuni* (Oh and Jeon, 2015). It would be likely that short LOS may easily expose the outer membrane of *C. jejuni* to hydrophobic phenolic compounds compared to long LPS structures. Taxifolin is an antioxidant that scavenges free radicals (Anthony and Saleh, 2013). Although the membrane permeabilizing effect of taxifolin has not been reported in bacteria, in this study, we also demonstrated that taxifolin significantly affects membrane permeability (**Figure 1C**), antibiotic accumulation (**Figure 2C**), and susceptibility in *C. jejuni* (**Table 1** and Supplementary Tables S1–S8). *p*-Coumaric acid is known as an antioxidant that decreases the peroxidation of low-density lipoprotein (LDL; Zang et al., 2000). Garrait et al. (2006) suggested that *p*-coumaric acid is a less metabolized phenolic antioxidant in rat model that may have greater health benefits. *p*-Coumaric acid possesses antimicrobial activities against Gram-positives and -negatives, and affects the membrane integrity in *Shigella dysenteriae* (Lou et al., 2012). In addition, *p*-coumaric acid binding to DNA to inhibit cellular functions that may cause cell death in *S. dysenteriae* (Lou et al., 2012). *p*-Coumaric acid significantly enhanced membrane permeability and drug accumulation in *C. jejuni* (**Figures 2A** and **3A**).

In some other bacteria, phenolic compounds may affect the expression of drug eﬄux pumps. Biochanin A, a phenolic compound from plants, exhibits an eﬄux pump inhibition activity in *Mycobacterium smegmatis* (Lechner et al., 2008). In contrast, exposure to a combination of phenolic compounds activates the expression of the *acrA* and *emrA* drug eﬄux pump genes in *Erwinia chrysanthemi*, a Gram-negative plant pathogen (Ravirala et al., 2007). In this study, some phenolic compounds reduced the expression level of CmeABC (**Figure 3**), an RND-type multidrug eﬄux pump that plays a key role in antimicrobial resistance in *C. jejuni* (Lin et al., 2002). Based on the findings in this study and reports from others, phenolic compounds may have differential, either negative or positive, effects on the expression of drug eﬄux pumps. Even though the reduced expression of CmeABC by phenolic compounds may result in the increased accumulation of ciprofloxacin (**Figures 2** and **3**), it cannot completely explain the synergistic effect of phenolic compounds. For example, *p*-coumaric acid significantly increased ciprofloxacin accumulation (**Figure 2A**) and exhibited substantial synergistic anti-*C. jejuni* activity with ciprofloxacin and erythromycin (**Table 1**) without affecting the protein level of CmeA (**Figure 3B**). Therefore, the increase in permeability and ciprofloxacin accumulation by *p*-coumaric acid might be related to the changes in membrane permeability and antibiotic influx. Taken together, the synergistic effects of phenolic compounds may involve at least two possible mechanisms: (1) phenolic compounds increase influx of antibiotics by permeabilizing the bacterial membrane, and (2) they inhibit the activity of the major eﬄux pump CmeABC. This hypothesis is supported by our findings that phenolic compounds increased the NPN accumulation even in the *cmeB* mutant where the function of CmeABC is inactivated (**Figure 4**).

The development of antimicrobial adjuvants that inhibit the function of resistance determinants is considered as a novel approach to curb antibiotic resistance (Wright, 2000), because this alternative strategy may re-sensitize pathogens to antibiotics and enhance the utility of existing antibiotics (Wright, 2000; Pages and Amaral, 2009). The findings in this study suggest that phenolic compounds could be used as a dietary adjuvant for antibiotic treatment of human infections with *Campylobacter*. Phenolic compounds are rich in foods of plant origin. For instance, one gram chestnut contains about 24.9 mg of gallic acid (Bennett et al., 2009) and one gram chokeberry has 1.4 mg of caffeic acid (Zheng and Wang, 2003). Since the site of *Campylobacter* infection is the gastrointestinal tracts, and antibiotics are generally administered orally for the treatment of foodborne infection with *Campylobacter*, possibly, the consumption of diets containing high levels of phenolic compounds may synergistically inhibit *Campylobacter* in the intestines. To prove this hypothesis, however, further experiments are required to examine the synergistic anti-*Campylobacter* effect *in vivo*, although the lack of suitable infection models for *Campylobacter* may be an issue for this. Nevertheless, our findings in this study successfully suggest the possibility that phenolic compounds may potentially be used for dietary therapy to treat human infections with *Campylobacter* with antibiotic resistance.

## Conflict of Interest Statement

The authors declare that the research was conducted in the absence of any commercial or financial relationships that could be construed as a potential conflict of interest.
