# Supplementary material for: Synergistic anti-Campylobacter jejuni activity of fluoroquinolone and macrolide antibiotics with phenolic compounds
Source: Front Microbiol. 2015 Oct 13;6:1129. doi: 10.3389/fmicb.2015.01129 (PMC4602130; doi:10.3389/fmicb.2015.01129)
Supplement: Supplementary file 1 [file Presentation_1.PDF]

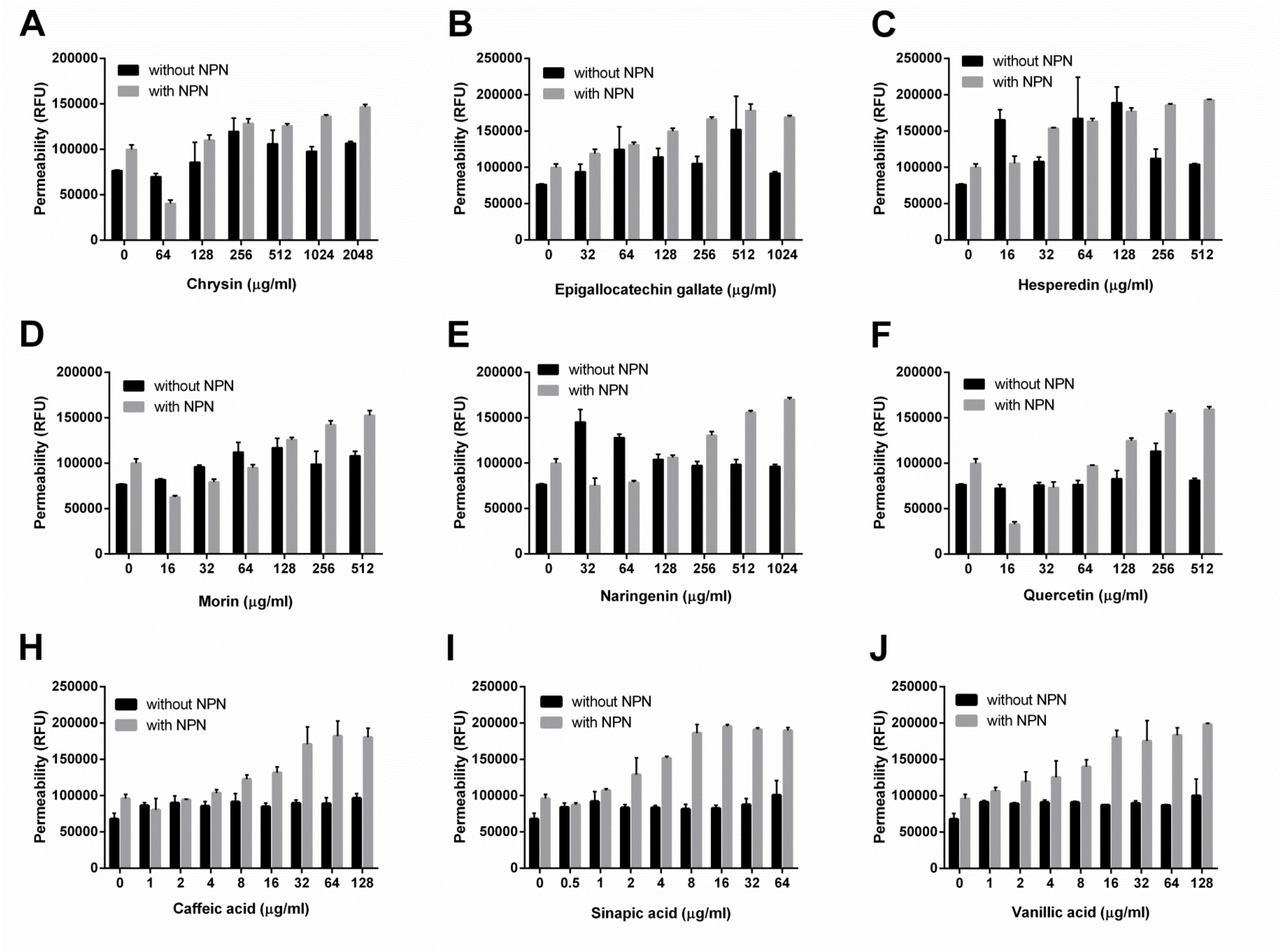

Fig. S1. Changes in the membrane permeability of *C. jejuni* NCTC 11168 by chrysin (A), epigallocatechin gallate (B), hesperedin (C), morin (D), naringenin (E), quercetin (F), caffeic acid (G), sinapic acid (H), and vanillic acid (I). The assay was performed with 10 μM 1-*N*-phenylnaphthylamine (NPN). The experiment was repeated three times, and all the experiments showed similar results.

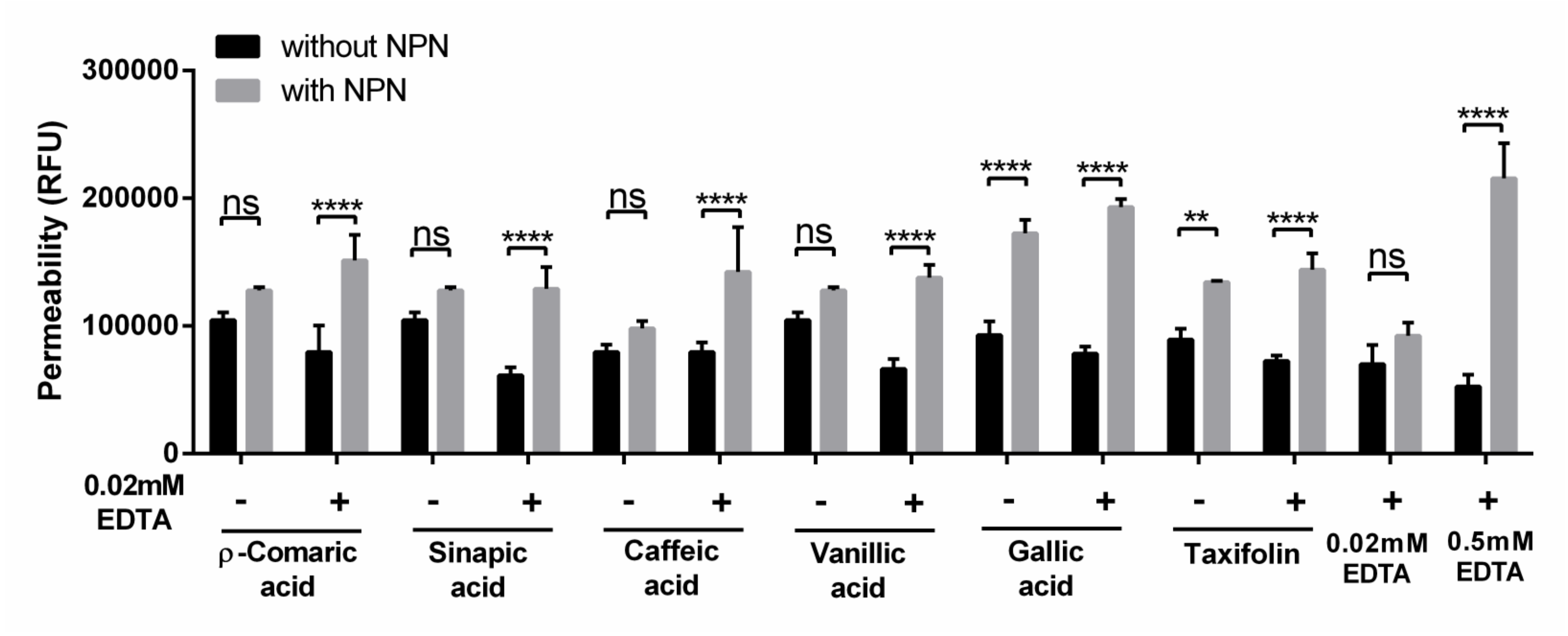

Fig. S2. Changes in the membrane permeability of *C. jejuni* NCTC 11168 by phenolic compounds. The concentration of phenolic compounds was 1μg/ml with or without EDTA. The assay was performed with 10 μM 1-*N*-phenylnaphthylamine (NPN). The experiment was repeated three times, providing similar results.

Table S1. Screening of phenolic compounds that exhibit synergistic anti-*C. jejuni* activity with ciprofloxacin.

Ciprofloxacin MIC : 0.5 μg ml^{-1}				
		Phenolics	256 μg ml^{-1} phenolics	MIC of phenolics
Phenolic acid†	Benzoic derivative	Salicylic acid	0.125 (4)	512
		Protocatechuic acid	0.5 (1)	1024
		Tannic acid	0.125 (4)	1024
		Benzoic acid	0.125 (4)	512
		ρ-Hydroxybenzoic acid	0.125 (4)	512
		Syringic acid	0.063 (8)	512
	Cinnamic derivative	Cinnamic acid	0.125 (4)	1024
		Ferulic acid	0.063 (8)	1024
Flavonoid†	Flavan-3-ol	Epigallocatechin gallate	0.063 (8)	512
		(-)-Epicatechin	0.125 (4)	1024
	Flavonol	Quercetin	-	256
		Morin hydrate	-	256
	Flavone	Chrysin	0.125 (4)	1024
	Flavonones	Naringenin	0.063 (8)	512
		Hesperedin	-	256

†The concentration of phenolic compounds is 256 μg ml^{-1}. The phenolic compounds listed in Table S1 are those without significant synergistic anti-*C. jejuni* activity. The phenolic compounds with synergistic effects are listed in Table 1.

Table S2. Screening of phenolic compounds that exhibit synergistic anti-*C. jejuni* activity with erythromycin.

Erythromycin MIC : 0.5 μg ml^{-1}				
		Phenolics	256 μg ml^{-1} phenolics	phenolics MIC
Phenolic acid†	Benzoic derivative	Salicylic acid	0.25 (2)	512
		Protocatechuic acid	0.25 (2)	1024
		Tannic acid	0.125 (4)	1024
		Benzoic acid	0.25 (2)	512
		ρ-Hydroxybenzoic acid	0.063 (8)	512
		Syringic acid	0.063 (8)	512
	Cinnamic derivative	Cinnamic acid	0.25 (2)	1024
		Ferulic acid	0.125 (4)	1024
Flavonoid†	Flavan-3-ol	Epigallocatechin gallate	0.063 (8)	512
		(-)-Epicatechin	0.063 (8)	1024
	Flavonol	Quercetin	-	256
		Morin hydrate	-	256
	Flavone	Chrysin	0.25 (2)	1024
	Flavonones	Naringenin	0.031 (16)	512
		Hesperedin	-	256

†The concentration of phenolic compounds is 256 μg ml^{-1}. The phenolic compounds listed in Table S2 are those without significant synergistic anti-*C. jejuni* activity. The phenolic compounds with synergistic effects are listed in Table 1.

Table S3 . Synergistic antimicrobial effect of ciprofloxacin and erythromycin with phenolics against *C. jejuni* NCTC11168.

Ciprofloxacin MIC (μg ml^{-1})						
Phenolics (μg ml^{-1})	*p*-Coumaric acid	Sinapic acid	Caffeic acid	Vanillic acid	Gallic acid	Taxifolin
256	-	0.008 (64)	0.016 (32)	0.008 (64)	0.008 (64)	-
128	0.008 (64)	0.031 (16)	0.031 (16)	0.008 (64)	0.016 (32)	0.008 (64)
64	0.016 (32)	0.031 (16)	0.031 (16)	0.016 (32)	0.031 (16)	0.031 (16)
32	0.016 (32)	0.063 (8)	0.063 (8)	0.031 (16)	0.031 (16)	0.031 (16)
16	0.063 (8)	0.063 (8)	0.063 (8)	0.063 (8)	0.031 (16)	0.031 (16)
8	0.063 (8)	0.063 (8)	0.125 (4)	0.063 (8)	0.063 (8)	0.063 (8)
4	0.125 (4)	0.063 (8)	0.125 (4)	0.125 (4)	0.063 (8)	0.063 (8)
2	0.125 (4)	0.063 (8)	0.125 (4)	0.125 (4)	0.063 (8)	0.063 (8)
0	0.5 (1)	0.5 (1)	0.5 (1)	0.5 (1)	0.5 (1)	0.5 (1)

Erythromycin MIC (μg ml^{-1})						
Phenolics (μg ml^{-1})	*p*-Coumaric acid	Sinapic acid	Caffeic acid	Vanillic acid	Gallic acid	Taxifolin
256	0.016 (32)	0.008 (64)	0.008 (64)	0.008 (64)	-	-
128	0.031 (16)	0.016 (32)	0.016 (32)	0.016 (32)	0.008 (64)	-
64	0.063 (8)	0.031 (16)	0.016 (32)	0.031 (16)	0.016 (32)	0.008 (64)
32	0.063 (8)	0.031 (16)	0.063 (8)	0.063 (8)	0.016 (32)	0.016 (32)
16	0.125 (4)	0.031 (16)	0.063 (8)	0.063 (8)	0.031 (16)	0.016 (32)
8	0.125 (4)	0.063 (8)	0.125 (4)	0.125 (4)	0.063 (8)	0.031 (16)
4	0.25 (2)	0.063 (8)	0.125 (4)	0.125 (4)	0.063 (8)	0.063 (8)
2	0.25 (2)	0.063 (8)	0.125 (4)	0.125 (4)	0.125 (4)	0.063 (8)
0	0.5 (1)	0.5 (1)	0.5 (1)	0.5 (1)	0.5 (1)	0.5 (1)

Table S4. Synergistic antimicrobial effect of ciprofloxacin and erythromycin with phenolics against *C. jejuni* P1, a poultry isolate.

	Ciprofloxacin MIC (μg ml^{-1})					
Phenolics (μg ml^{-1})	ρ-Coumaric acid	Sinapic acid	Caffeic acid	Vanillic acid	Gallic acid	Taxifolin
256	0.002 (64)	0.004 (32)	0.008 (16)	0.004 (32)	0.002 (64)	0.002 (64)
128	0.002 (64)	0.008 (16)	0.004 (32)	0.008 (16)	0.004 (32)	0.002 (64)
64	0.002 (64)	0.008 (16)	0.004 (32)	0.008 (16)	0.004 (32)	0.004 (32)
32	0.002 (64)	0.004 (16)	0.004 (32)	0.008 (16)	0.004 (32)	0.004 (32)
16	0.002 (64)	0.008 (16)	0.008 (16)	0.008 (16)	0.004 (32)	0.008 (16)
8	0.004 (32)	0.016 (8)	0.031 (4)	0.008 (16)	0.008 (16)	0.008 (16)
4	0.004 (32)	0.016 (8)	0.031 (4)	0.008 (16)	0.016 (8)	0.008 (16)
2	0.004 (32)	0.031 (4)	0.031 (4)	0.008 (16)	0.016 (8)	0.016 (8)
0	0.125 (1)	0.125 (1)	0.125 (1)	0.125 (1)	0.125 (1)	0.125 (1)

	Erythromycin MIC (μg ml^{-1})					
Phenolics (μg ml^{-1})	ρ-Coumaric acid	Sinapic acid	Caffeic acid	Vanillic acid	Gallic acid	Taxifolin
256	0.016 (32)	0.008 (64)	0.016 (32)	0.031 (16)	0.016 (32)	0.008 (64)
128	0.016 (32)	0.016 (32)	0.016 (32)	0.031 (16)	0.031 (16)	0.016 (32)
64	0.031 (16)	0.016 (32)	0.031 (16)	0.063 (8)	0.031 (16)	0.016 (32)
32	0.031 (16)	0.031 (16)	0.031 (16)	0.063 (8)	0.063 (8)	0.016 (32)
16	0.031 (16)	0.063 (8)	0.063 (8)	0.125 (4)	0.063 (8)	0.031 (16)
8	0.063 (8)	0.063 (8)	0.063 (8)	0.125 (4)	0.125 (4)	0.031 (16)
4	0.063 (8)	0.125 (4)	0.125 (4)	0.25 (2)	0.125 (4)	0.063 (8)
2	0.125 (4)	0.125 (4)	0.125 (4)	0.25 (2)	0.125 (4)	0.063 (8)
0	0.5 (1)	0.5 (1)	0.5 (1)	0.5 (1)	0.5 (1)	0.5 (1)

Table S5. Synergistic antimicrobial effect of ciprofloxacin and erythromycin with phenolics against *C. jejuni* P2, a poultry isolate.

	Ciprofloxacin MIC (μg ml^{-1})					
Phenolics (μg ml^{-1})	ρ-Coumaric acid	Sinapic acid	Caffeic acid	Vanillic acid	Gallic acid	Taxifolin
256	0.031 (32)	0.016 (64)	0.031 (32)	0.031 (32)	0.016 (64)	0.016 (64)
128	0.031 (32)	0.031 (32)	0.031 (32)	0.063 (16)	0.031 (32)	0.031 (32)
64	0.063 (16)	0.031 (32)	0.063 (16)	0.063 (16)	0.031 (32)	0.031 (32)
32	0.063 (16)	0.031 (32)	0.063 (16)	0.063 (16)	0.031 (32)	0.031 (32)
16	0.063 (16)	0.063 (16)	0.125 (8)	0.125 (8)	0.063 (16)	0.063 (16)
8	0.125 (8)	0.063 (16)	0.125 (8)	0.25 (4)	0.125 (8)	0.063 (16)
4	0.125 (8)	0.125 (8)	0.25 (4)	0.25 (4)	0.125 (8)	0.125 (8)
2	0.25 (4)	0.125 (8)	0.5 (2)	0.5 (2)	0.125 (8)	0.125 (8)
0	1 (1)	1 (1)	1 (1)	1 (1)	1 (1)	1 (1)

	Erythromycin MIC (μg ml^{-1})					
Phenolics (μg ml^{-1})	ρ-Coumaric acid	Sinapic acid	Caffeic acid	Vanillic acid	Gallic acid	Taxifolin
256	0.016 (64)	0.031 (32)	0.063 (16)	0.031 (32)	0.016 (64)	0.016 (64)
128	0.031 (32)	0.031 (32)	0.063 (16)	0.063 (16)	0.031 (32)	0.016 (64)
64	0.031 (32)	0.063 (16)	0.125 (8)	0.063 (16)	0.063 (16)	0.031 (32)
32	0.063 (16)	0.063 (16)	0.125 (8)	0.125 (8)	0.063 (16)	0.031 (32)
16	0.063 (16)	0.125 (8)	0.125 (8)	0.125 (8)	0.063 (16)	0.063 (16)
8	0.125 (8)	0.125 (8)	0.25 (4)	0.25 (4)	0.125 (8)	0.063 (16)
4	0.25 (4)	0.25 (4)	0.25 (4)	0.25 (4)	0.125 (8)	0.125 (8)
2	0.25 (4)	0.5 (2)	0.5 (2)	0.5 (2)	0.125 (8)	0.125 (8)
0	1 (1)	1 (1)	1 (1)	1 (1)	1 (1)	1 (1)

Table S6. Synergistic antimicrobial effect of ciprofloxacin and erythromycin with phenolics against *C. jejuni* HCJ4132, a human isolate.

	Ciprofloxacin MIC (μg ml^{-1})					
Phenolics (μg ml^{-1})	ρ-Coumaric acid	Sinapic acid	Caffeic acid	Vanillic acid	Gallic acid	Taxifolin
256	0.004 (64)	0.004 (64)	0.008 (32)	0.008 (32)	0.004 (64)	0.008 (32)
128	0.004 (64)	0.004 (64)	0.008 (32)	0.008 (32)	0.008 (32)	0.008 (32)
64	0.008 (32)	0.008 (32)	0.008 (32)	0.016 (16)	0.008 (32)	0.008 (32)
32	0.008 (32)	0.016 (16)	0.016 (16)	0.016 (16)	0.016 (16)	0.016 (16)
16	0.016 (16)	0.016 (16)	0.031 (8)	0.031 (8)	0.016 (16)	0.031 (8)
8	0.016 (16)	0.031 (8)	0.031 (8)	0.031 (8)	0.016 (16)	0.031 (8)
4	0.031 (8)	0.031 (8)	0.063 (4)	0.031 (8)	0.031 (8)	0.031 (8)
2	0.063 (4)	0.031 (8)	0.063 (4)	0.063 (4)	0.063 (4)	0.031 (8)
0	0.25 (1)	0.25 (1)	0.25 (1)	0.25 (1)	0.25 (1)	0.25 (1)

	Erythromycin MIC (μg ml^{-1})					
Phenolics (μg ml^{-1})	ρ-Coumaric acid	Sinapic acid	Caffeic acid	Vanillic acid	Gallic acid	Taxifolin
256	0.031 (8)	0.016 (16)	0.031 (8)	0.063 (4)	0.016 (16)	0.008 (32)
128	0.031 (8)	0.031 (8)	0.031 (8)	0.063 (4)	0.016 (16)	0.016 (16)
64	0.063 (4)	0.031 (8)	0.063 (4)	0.063 (4)	0.031 (8)	0.016 (16)
32	0.063 (4)	0.063 (4)	0.063 (4)	0.125 (2)	0.031 (8)	0.031 (8)
16	0.125 (2)	0.125 (2)	0.063 (4)	0.125 (2)	0.031 (8)	0.031 (8)
8	0.25 (1)	0.125 (2)	0.125 (2)	0.125 (2)	0.063 (4)	0.063 (4)
4	0.25 (1)	0.125 (2)	0.125 (2)	0.25 (1)	0.063 (4)	0.063 (4)
2	0.25 (1)	0.25 (1)	0.125 (2)	0.25 (1)	0.063 (4)	0.125 (2)
0	0.25 (1)	0.25 (1)	0.25 (1)	0.25 (1)	0.25 (1)	0.25 (1)

Table S7. Synergistic antimicrobial effect of ciprofloxacin and erythromycin with phenolics against *C. jejuni* HCJ2316, a human isolate.

	Ciprofloxacin MIC (μg ml^{-1})					
Phenolics (μg ml^{-1})	ρ-Coumaric acid	Sinapic acid	Caffeic acid	Vanillic acid	Gallic acid	Taxifolin
256	0.004 (64)	0.004 (64)	0.008 (32)	0.008 (32)	0.004 (64)	0.008 (32)
128	0.004 (64)	0.004 (64)	0.008 (32)	0.008 (32)	0.008 (32)	0.008 (32)
64	0.008 (32)	0.008 (32)	0.008 (32)	0.016 (16)	0.008 (32)	0.008 (32)
32	0.008 (32)	0.016 (16)	0.016 (16)	0.016 (16)	0.016 (16)	0.016 (16)
16	0.016 (16)	0.016 (16)	0.031 (8)	0.031 (8)	0.031 (8)	0.031 (8)
8	0.016 (16)	0.031 (8)	0.031 (8)	0.031 (8)	0.031 (8)	0.031 (8)
4	0.031 (8)	0.031 (8)	0.063 (4)	0.031 (8)	0.031 (8)	0.031 (8)
2	0.063 (4)	0.031 (8)	0.063 (4)	0.063 (4)	0.063 (4)	0.031 (8)
0	0.25 (1)	0.25 (1)	0.25 (1)	0.25 (1)	0.25 (1)	0.25 (1)

	Erythromycin MIC (μg ml^{-1})					
Phenolics (μg ml^{-1})	ρ-Coumaric acid	Sinapic acid	Caffeic acid	Vanillic acid	Gallic acid	Taxifolin
256	0.008 (32)	0.016 (16)	0.008 (64)	0.016 (16)	0.008 (64)	0.008 (64)
128	0.016 (16)	0.016 (16)	0.031(8)	0.031(8)	0.016 (16)	0.008 (64)
64	0.016 (16)	0.016 (16)	0.031(8)	0.031(8)	0.016 (16)	0.016 (16)
32	0.031(8)	0.031(8)	0.063 (4)	0.063 (4)	0.031(8)	0.016 (16)
16	0.031(8)	0.031(8)	0.063 (4)	0.063 (4)	0.031(8)	0.031(8)
8	0.063 (4)	0.063 (4)	0.125 (2)	0.125 (2)	0.063 (4)	0.031(8)
4	0.125 (2)	0.063 (4)	0.125 (2)	0.125 (2)	0.063 (4)	0.063 (4)
2	0.125 (2)	0.25 (1)	0.25 (1)	0.25 (1)	0.125	0.063 (4)
0	0.25 (1)	0.25 (1)	0.25 (1)	0.25 (1)	0.25 (1)	0.25 (1)

Table S8. Synergistic antimicrobial activity of phenolic compounds against *C. jejuni* strains resistant to ciprofloxacin and erythromycin

	Ciprofloxacin MIC (μg ml^{-1})						
	Phenolics (μg ml^{-1})	*p*-Coumaric acid	Sinapic acid	Caffeic acid	Vanillic acid	Gallic acid	Taxifolin
C. jejuni CR64	256	-	2 (32)	-	-	-	-
	128	1 (64)	4 (16)	1 (64)	2 (32)	-	-
	64	2 (32)	4 (16)	4 (16)	2 (32)	1 (64)	1 (64)
	32	4 (16)	4 (16)	8 (8)	4 (16)	2 (32)	1 (64)
	16	8 (8)	8 (8)	8 (8)	8 (8)	4 (16)	2 (32)
	8	8 (8)	8 (8)	16 (4)	8 (8)	8 (8)	2 (32)
	4	16 (4)	16 (4)	16 (4)	16 (4)	8 (8)	8 (8)
	2	32 (2)	16 (4)	32 (2)	16 (4)	16 (4)	16 (4)
	0	64 (1)	64 (1)	64 (1)	64 (1)	64 (1)	64 (1)

	Erythromycin MIC (μg ml^{-1})						
	Phenolics (μg ml^{-1})	*p*-Coumaric acid	Sinapic acid	Caffeic acid	Vanillic acid	Gallic acid	Taxifolin
C. jejuni ER641	256	1 (64)	1 (64)	1 (64)	1 (64)	1 (64)	-
	128	2 (32)	2 (32)	2 (32)	2 (32)	2 (32)	-
	64	4 (16)	4 (16)	4 (16)	4 (16)	4 (16)	1 (64)
	32	8 (8)	4 (16)	8 (8)	4 (16)	4 (16)	2 (32)
	16	16 (4)	8 (8)	16 (4)	8 (8)	8 (8)	4 (16)
	8	16 (4)	16 (4)	16 (4)	16 (4)	16 (4)	4 (16)
	4	32 (2)	32 (2)	32 (2)	16 (4)	32 (2)	8 (8)
	2	64 (1)	32 (2)	64 (1)	32 (2)	32 (2)	16 (4)
	0	64 (1)	64 (1)	64 (1)	64 (1)	64 (1)	64 (1)

C. jejuni CR64 and ER641 are *C. jejuni* NCTC11168 derivatives resistant to ciprofloxacin and erythromycin, respectively.

Table S9. FIC index of combinational treatment of *C. jejuni* NCTC 11168 with antibiotics and phenolic compounds.

Ciprofloxacin with phenolics						
Phenolics (μg ml^{-1})	*p*-Coumaric acid	Sinapic acid	Caffeic acid	Vanillic acid	Gallic acid	Taxifolin
256	-	0.516	0.266	0.266	0.516	-
128	0.141	0.312	0.157	0.141	0.282	0.516
64	0.095	0.187	0.095	0.095	0.187	0.312
32	0.063	0.189	0.157	0.093	0.125	0.187
16	0.141	0.157	0.142	0.142	0.093	0.125
8	0.134	0.147	0.258	0.134	0.142	0.157
4	0.254	0.134	0.254	0.254	0.134	0.142
2	0.252	0.13	0.252	0.252	0.13	0.134
0	1	1	1	1	1	1
**Erythromycin with phenolics**						
Phenolics (μg ml^{-1})	*p*-Coumaric acid	Sinapic acid	Caffeic acid	Vanillic acid	Gallic acid	Taxifolin
256	0.282	0.516	0.266	0.266	-	-
128	0.187	0.282	0.157	0.157	0.266	-
64	0.189	0.187	0.095	0.124	0.157	0.266
32	0.157	0.125	0.157	0.157	0.094	0.157
16	0.266	0.093	0.142	0.141	0.093	0.094
8	0.258	0.142	0.258	0.257	0.141	0.093
4	0.503	0.134	0.254	0.253	0.133	0.141
2	0.502	0.13	0.252	0.251	0.253	0.133
0	1	1	1	1	1	1

The combinations correspond to Table S3. FIC index of combinational treatment of *C. jejuni* NCTC 11168 with antibiotics and phenolic

compounds. To evaluate synergism between antibiotics and phenolics, FIC index was calculated according to a method described by Rand *et al*. (1): FIC index = (MIC of antibiotic and phenolic combination)/(MIC antibiotic alone) + (MIC of combination antibiotic and phenolic combination)/(MIC of phenolic alone). FIC index of 0.5 or less indicates synergism.

Reference: 1. Rand K, Houck H, Brown P, Bennett D. 1993. Reproducibility of the microdilution checkerboard method for antibiotic synergy. Antimicrob. Agents Chemother. **37:**613-615.
